# A national population-based study of mortality and risk factors in COVID-19-hospitalized patients in Spain (2020–2021)

**DOI:** 10.3389/fpubh.2025.1488283

**Published:** 2025-02-04

**Authors:** José-Manuel Ramos-Rincón, José Sánchez-Paya, Pilar González-De-La-Aleja, Juan-Carlos Rodríguez-Díaz, Esperanza Merino

**Affiliations:** ^1^Department of Internal Medicine, Alicante Institute for Health and Biomedical Research (ISABIAL), Dr. Balmis General University Hospital, Alicante, Spain; ^2^Miguel Hernández University of Elche, Alicante, Spain; ^3^Preventive Service, Alicante Institute for Health and Biomedical Research (ISABIAL), Dr. Balmis General University Hospital, Alicante, Spain; ^4^Unit of Infectious Diseases, Alicante Institute for Health and Biomedical Research (ISABIAL), Dr. Balmis General University Hospital, Alicante, Spain; ^5^Service of Microbiology, Alicante Institute for Health and Biomedical Research (ISABIAL), Dr. Balmis General University Hospital, Alicante, Spain

**Keywords:** COVID-19, SARS-CoV-2, Spain, hospitalization, risk factor mortality, in-hospital mortality, vaccination

## Abstract

**Objectives:**

The study aimed to analyze in-hospital mortality (IHM) among all COVID-19 patients hospitalized in Spain between March 1, 2020, and December 31, 2021, and to compare two distinct periods: the prevaccination period (March 1, 2020, to January 31, 2021) and the vaccination period (February 1, 2021, to December 31, 2021). The objective was to assess the impact of vaccination on IHM and identify associated risk factors, using data from Spain’s national hospitalization registry.

**Methods:**

This retrospective analysis used data from the Spanish National Surveillance System for Hospital Data. The primary outcome was in-hospital mortality (IHM). Multivariate logistic regression identified risk factors across the overall study period, as well as during the prevaccination and vaccination periods. Risk factors included age (in 20-year intervals), sex, comorbidities (e.g., hypertension, diabetes, chronic kidney failure, obesity, neurodegenerative disorders, and others), and admission to the intensive care unit.

**Results:**

A total of 524,314 COVID-19 hospitalizations were recorded in Spain, with 329,690 during the prevaccination period and 194,624 during the vaccination period. Hospitalization rates dropped from 697/100,000 people to 411/100,000, and in-hospital mortality (IHM) decreased from 16.2 to 11.5% (adjusted odds ratio [AOR]: 0.71, 95% CI: 0.70–0.73, *p* < 0.001). IHM rose with age, from 0.8% in patients aged 18–39 to 31.7% in those ≥80 years (*p* < 0.001), but significant decreases were observed across all age groups after vaccination, especially in those ≥80 years (AOR: 0.76, 95% CI: 0.75–0.79, *p* < 0.001). Risk factors for IHM remained consistent, with leukemia, neoplasm, and lymphoma posing the highest risks, while female sex (AOR: 0.75, 95% CI: 0.74–0.77, *p* < 0.001) and dyslipidemia (AOR: 0.85, 95% CI: 0.32–0.86, *p* < 0.001) were protective factors.

**Conclusion:**

During the vaccination period, the risk of in-hospital mortality (IHM) was 29% lower than in the prevaccination period, after adjusting for sex, age, and comorbidities. This reduced risk was observed across sexes, age groups, and comorbidities. The risk factors for IHM remained consistent between the two periods, with age as the main risk factor, while female sex and dyslipidemia were identified as protective factors.

## 1 Introduction

Since the emergence of the SARS-CoV-2 pandemic in December 2019 until October 2023, it has caused more than 676 million cases and more than 6.88 million deaths worldwide (1). In Spain, one of the most affected countries in the European Union, there have been more than 13.7 million cases and a total of 119,470 deaths (1). During the first 2 years, COVID-19 caused high mortality (2, 4). Numerous risk factors have been consistently associated with COVID-19 mortality, advanced age remains one of the most significant predictors, with individuals over 60 years, and particularly those over 80 years, facing a markedly higher risk of death (5, 6). Comorbidities, such as hypertension, diabetes, cardiovascular diseases, chronic kidney disease, chronic obstructive pulmonary disease (COPD), malignancy, and immunosuppression have been shown to further exacerbate mortality risk (2, 4, 6, 7). Moreover, patients requiring admission to intensive care units (ICU) exhibit an even more pronounced risk of death (4, 6–8). Additionally, female sex has been associated with a lower risk of COVID-19-related mortality (6, 7, 9, 10). Furthermore, vaccination has been shown to significantly reduce hospital admissions and mortality in various studies conducted in other countries (11–13).

COVID-19 pandemic has significantly impacted Spain, with the country experiencing some of the highest incidence and mortality rates in Europe (2, 4, 5, 9). During the initial phase of the pandemic, Spain faced six waves of infection up to January 2022, with the fifth wave being driven by the Delta variant and characterized by a high incidence of cases (14). Vaccination campaign in Spain began in December 2020, and by the end of the fifth wave, 77.3% of the population had been fully vaccinated (14). The pre-vaccination period saw high rates of hospitalizations and mortality. The vaccination period showed a marked improvement in patient outcomes. The rapid vaccination rollout, prioritizing older adults and high-risk groups, resulted in a significant decline in COVID-19 hospitalizations and deaths starting in February 2021 (3). The vaccination campaign’s success is further supported by data indicating that fully vaccinated patients had less severe forms of COVID-19, and a higher probability of earlier discharge compared to unvaccinated patients (11). Moreover, vaccination was associated with a 20% reduction in the risk of case-fatality among hospitalized patients (15).

This study, based on data from the Spanish National Surveillance System for Hospital Data (SNSSHD) from the National Healthcare Service (NHS), aims to evaluate in-hospital mortality (IHM) before and after the implementation of the COVID-19 vaccination program. Despite extensive research on COVID-19, there remains a significant gap in the literature regarding the analysis of risk factors for IHM during the prevaccination and vaccination periods using a large-scale, national database. This study seeks to address this gap by investigating whether the risk factors for IHM have shifted between prevaccination and vaccination in Spain, offering valuable insights into the changing dynamics of the pandemic and the impact of vaccination on high-risk population. The use of observational approaches, such as retrospective analysis of registries or computerized healthcare utilization databases like SNSSHD, can quickly provide data from many patients (16, 17). Although these methods may introduce bias, they are useful for understanding rapid approaches (16).

The objectives of this study were: (1) to analyze in-hospital mortality (IHM) among all hospitalized COVID-19 patients in Spain between March 1, 2020, and December 31, 2021, and (2) to compare two distinct periods—the pre-vaccination period (March 1, 2020, to January 31, 2021) and the vaccination period (February 1, 2021, to December 31, 2021)—to assess the impact of vaccination on IHM and associated risk factors.

## 2 Methods

### 2.1 Data sources

This is an observational, transversal, and nationwide study using an administrative database, SNSSHD from the NHS, which contains all records of hospitalizations (95–97% of discharges) in all public and private hospitals in Spain. which includes the demographic characteristics of hospitalized patients (18). Researchers have used this database to analyze the epidemiological and clinical aspects of numerous conditions. The SNSSHD allows for the exploration of various populational and epidemiological aspects of the COVID-19 pandemic in Spain. Previous studies have utilized this database to investigate different epidemiological aspects during the first and second years of the pandemic, including the prevalence and cost of hospitalized patients with asymptomatic COVID-19, the epidemiology of bacterial coinfections and risk factors in hospitalized patients with COVID-19, and the impact of environmental factors on hospital outcomes during and after the lockdown in 2020 (18–22).

We extracted all hospital admission of people ≥18 years old at the time of hospital admission that occurred between 1 March 2020 and 31 December 2021. The data collected included information on the discharge date of each hospitalization. Cases were as follows: (1) From March 1, 2020, to June 30, 2020, the codes included “B34.2,” “B97.29,” “Z20.828,” “J12.82,” “J12.81,” and “U07.1”; and (2) from July 1, 2020, to December 31, 2021, only the code “U07.1” was used. The total Spanish population as of January 1, 2020, and January 1, 2021, was obtained from the National Statistical Institute (23).

### 2.2 Variables

The variables collected for each hospitalization episode were age, sex, comorbidities, admission to the intensive care unit (ICU), and in-hospital mortality. Age groups were categorized according to 20-year intervals: 18–39, 40–59, 60–79, and ≥ 80 years. Comorbidities were extracted via ICD-10 diagnostic codes, the discharge date (up to 20 diagnoses) and classified into the following categories: hypertension, dyslipidemia, diabetes mellitus, chronic kidney failure, obesity, neurodegenerative disorder (including dementia), heart failure, ischemic heart disease, COPD, cerebrovascular disease, hemodialysis, transplantation, chronic liver disease, leukemia, and human immunodeficiency virus (HIV) infection (Supplementary Table S1). In this administrative database, each hospital admission is considered a new patient, and readmissions are treated as separate cases.

The prevaccination period covered the wave in which the D614G SARS-CoV-2 variant was dominant and included patients diagnosed with COVID-19 between 1 March 2020 and 31 January 2021 (24). The vaccination period covered the alpha- and delta-dominant waves and included patients diagnosed between February 1 and December 31, 2021. Vaccination in Spain began on December 27, 2020, and by August 2021, 75% of the population had been vaccinated (25). Although vaccination began on December 27, we considered the pre-vaccination period to extend until January 31 because, during the early days of the vaccination campaign, only specific groups—such as the older adults, healthcare workers, and immunocompromised individuals—received a single dose. These groups did not begin receiving the second dose until February, and the broader population was not significantly reached until later. This is the rationale behind our decision.

### 2.3 Statistical analysis

The rates of COVID-19-related hospitalization were calculated per 100,000 people stratified by prevaccination and vaccination study periods and sex. All of variables were categorical variables are expressed as absolute values and percentages. Bivariable comparisons of qualitative variables were performed via the chi-square test. All tests were two-tailed, and only *p*-values of less than 0.05 were considered significant.

The measures of association are presented as odds ratios (ORs) with 95% confidence intervals (CIs). In the comparison of IHM between the prevaccination and vaccination study periods, the vaccination period was considered the exposure factor for calculating the ORs. In the analysis of risk factors for overall IHM, as well as during the pre-vaccination and vaccination periods, the exposure factors included: vaccination status, female sex, age groups (40–59, 60–79, and > 80 years), and the presence of the following comorbidities or conditions: hypertension, dyslipidemia, diabetes mellitus, obesity, chronic kidney failure, neurodegenerative diseases, heart failure, ischemic heart disease, COPD, neoplasm, cerebrovascular disease, lymphoma, organ transplant, hemodialysis, chronic liver disease, leukemia, HIV infection, and ICU admission.

Multivariate logistic regression analysis was used to identify independent predictors of IHM in the prevaccination and vaccination study periods. Variables with a *p* value below 0.05 in the univariate analyses were entered into a multivariate logistic regression model using a stepwise selection method with the likelihood ratio test. The measures of association after multivariate analysis were presented adjusted OR (AOR) with 95% CIs. All the statistical analyses were performed via the IBM SPSS package for Windows v25.0 (IBM Corp, Armonk, NY).

### 2.4 Ethics statement

This study involves the use of medical data from the SNSSHD for the NHS. To guarantee patients’ anonymity, the database was provided to us by the NHS after the removal of all potential patient identifiers. The procedures described here were carried out in accordance with the ethical standards described in the Revised Declaration of Helsinki in 2013.

## 3 Results

### 3.1 Rates of COVID-19-related hospitalization in prevaccination and vaccination period by sex and age

During the study period, 524,314 hospitalizations related to COVID-19 were registered in Spain. In the prevaccination period, there were 329,690 admissions and 194,624 in the vaccination study period (Table 1). During the prevaccination period, the hospitalization rate was 697 per 100,000 of the overall population, with 783 per 100,000 for males and 613 per 100,000 for females. In contrast, during the vaccination period, the hospitalization rate decreased significantly to 411 per 100,000 overall, with rates of 475 per 100,000 in males and 347 per 100,000 in females (Table 1).

**Table 1:** Number of hospitalization and hospitalization rate per 100,000 habitants by sex and age.

	Overall period	Prevaccination period	Vaccination period
Hospitalization			
Total	524,314	329,690	194,624
Sex			
Male	292,376	181,765	110,611
Female	13,934	147,922	84,012
Age, range (years)			
18–39	48,398	24,071	24,327
40–59	139,196	82,827	56,369
60–79	199,945	126,673	72,784
≥80	48,398	24,071	24,327
Hospitalization rate per 100,000 habitants^a^			
Total	553	697	411
Sex			
Male	629	783	475
Female	480	613	347
Age, range (years)			
18–39	202	202	202
40–59	465	552	378
60–79	1,063	1,338	783
≥80	1,555	2,168	936

a The total Spanish population as of January 1, 2020, and January 1, 2021, total sex and age was obtained from the National Statistical Institute; The overall period is calculate by median of prevaccination period and vaccination period.

By age group, during the prevaccination period, the hospitalization rate was 202 per 100,000 in the 18–39 age group, 552 per 100,000 in the 40–59 age group, 1,338 per 100,000 in the 60–79 age group, and 2,168 per 100,000 in the 80+ age group. During the vaccination period, the hospitalization rate remained unchanged in the 18–39 age group (202 per 100,000) but declined significantly in the other groups: 378 per 100,000 in the 40–59 age group, 783 per 100,000 in the 60–79 age group, and 936 per 100,000 in the 80+ age group (Table 1).

### 3.2 Clinical variables across the two study periods

Table 2 presents the differences in the epidemiological and clinical variables between the prevaccination and vaccination study periods. The percentage of hospitalized males was greater in both study periods (55.1 and 56.8%). During the vaccination period, there was an increase in hospitalizations among younger age groups, specifically those aged 18--39 years and 40--59 years, increasing from 7.3 and 25.1% in the prevaccination period to 12.5 and 29.0% in the vaccination period, respectively. Conversely, the proportion of hospitalized individuals aged ≥80 years significantly decreased from 29.2% in the prevaccination period to 21.1% in the vaccination period (*p*-value <0.001).

**Table 2:** Differences in age, sex, comorbidities, admission to the intensive care unit, and in-hospital mortality by period of study.

	Overall period; *N* = 524,314; % (N)	Prevaccination period; *N* = 329,690; % (N)	Vaccination period; *N* = 194,624; % (N)	*p*-value*
Sex				
Male	55.8 (292,376)	55.1 (181,765)	56.8 (110,611)	<0.001
Female	44.2 (213,934)	44.9 (147,922)	43.2 (84,012)	
Age, range (years)				
18–39	9.2 (48,398)	7.3 (24,071)	12.5 (24,327)	<0.001
40–59	26.5 (139,196)	25.1 (82,827)	29.0 (56,369)	
60–79	28.0 (199,945)	38.4 (126,673)	37.4(72,784)	
≥80	26.2 (137,263)	29.2 (96,119)	21.1(41,144)	
Comorbidities and complications				
Hypertension	46.7 (244,771)	48.5(160,022)	43.5 (84,749)	<0.001
Dyslipidemia	30.0 (157,069)	30.9 (101,807)	28.4 (55,262)	<0.001
Diabetes mellitus	22.5 (117,715)	22.9 (75,537)	21.7 (42,178)	<0.001
Obesity	12.8 (67,114)	11.2 (37,077)	15.4 (30,037)	<0.001
CKF	10.7 (56,089)	11.3 (37,215)	9.7 (18,874)	<0.001
NDDs	7.4 (38,770)	8.5 (28,152)	5.5 (10,618)	<0.001
Heart failure	7.7 (40,553)	8.2 (26,991)	7.0 (13,562)	<0.001
IHD	7.2 (37,759)	7.6 (25,085)	6.5 (12,674)	<0.001
COPD	6.0 (31,289)	6.2 (20,578)	5.5 (10,711)	<0.001
Neoplasm	4.3 (22,744)	4.6 (15,130)	3.9 (7,614)	<0.001
CVD	2.9 (15,351)	3.2 (10,427)	2.5 (4,924)	<0.001
Lymphoma	1.4 (7,568)	1.4 (4,616)	1.5 (2,952)	<0.001
Transplant	0.8 (4,109)	0.7 (2,399)	0.9(1710)	<0.001
Hemodialysis	0.7 (3,528)	0.8 (2,478)	0.5 (1,050)	<0.001
CLD	0.4 (2,196)	0.4 (1,398)	0.4 (798)	0.448
Leukemia	0.2 (1,183)	0.3 (831)	0.2(352)	<0.001
HIV	0.2 (1,297)	0.2 (800)	0.3(497)	0.371
Outcome				
ICU admission	10.5 (55,224)	8.5 (28,099)	13.9 (27,125)	<0.001
IHM	14.4 (75,595)	16.2 (53,304)	11.5 (22,291)	<0.001

*The statistical test used was the chi-square test. %, percentage; N, number; IHM, in-hospital mortality; CKF, chronic kidney failure; IHD, ischemic heart disease; CVD, cerebrovascular disease; CLD, chronic liver disease; HIV, human immunodeficiency virus; NDDs, neurodegenerative disorders; COPD, chronic obstructive pulmonary disease.

In terms of the comorbidities evaluated, the prevalence of most conditions, including hypertension, dyslipidemia, diabetes mellitus, chronic kidney failure, neurodegenerative disorders, heart failure, ischemic heart disease, COPD, cerebrovascular disease, hemodialysis, and leukemia, significantly decreased in the vaccination period (*p*-value <0.001 for all). However, the prevalence of obesity, lymphoma, and transplant cases increased significantly (*p*-value <0.001).

The number of ICU admissions was notably greater among the 60–79 age group (15.4%) and lower among females (7.5% vs. 12.9% in males, *p*-value <0.001) and the older population (aged >80 years) at 1.8% (*p*-value <0.001). Admissions to the ICU increased from 8.5% in the prevaccination period to 13.9% in the vaccination period (*p*-value <0.001). This statistically significant increase was observed across both sexes and all age groups, as shown in Table 3. The largest percentage increases, all statistically significant (*p*-value <0.001), were seen in the 60–79 age group (7.2%), the 40–59 age group (5.7%), and among males (5.9%), while the smallest increase was observed in aged >80 years (0.8%).

**Table 3:** Frequency of hospitalizations with intensive care unit admission stratified by age and sex by period of study.

	Overall period; % (n/N)^a^	Prevaccination period; % (n/N)	Vaccination period; % (n/N)	*p*-value*
Overall	10.5 (55,224/523,937)	8.5 (28,099/329,632)	13.9 (27,125/194,305)	<0.001
Sex				
Male	12.9 (37,772/29,2,158)	10.7 (19,441/18,738)	16.6 (18,361/110,420)	<0.001
Female	7.5 (17,452/214327)	5.9 (8,688/147,894)	10.4 (8,764/17,452)	<0.001
Age, range (years)				
18–39	8.4 (4,073/48,328)	6.5 (1,570/24,066)	10.3 (2,503/24,262)	<0.001
40–59	12.8 (17,846/139,078)	10.5 (8,720/82,817)	16.2 (9,126/56,261)	<0.001
60–79	15.4 (30,771/199,334)	12.8 (16,257/126,648)	20.0 (14,514/72,686)	<0.001
≥80	1.8 (2,534/137,201)	1.6 (1,552/96,104)	2.4 (982/41,097)	<0.001

a The results are presented as percentages (number of events per sample size).

*The statistical test used was the chi-square test. %, percentage; n. number of cases, N, total number.

### 3.3 Mortality by clinical variables across the prevaccination and vaccination study periods

The overall IHM during the study period was 14.4%. A comparison of the IHM between the prevaccination and vaccination periods is shown in Table 4. In the prevaccination period, the IHM was 16.2%, which decreased to 11.5% during the vaccination period (exposure; OR: 0.67, 95% CI: 0.66–0.68, *p*-value <0.001).

**Table 4:** Comparison of in-hospital mortality (IHM) during the prevaccination and vaccination periods.

Variables	IHM during prevaccination period; % (n/N)^a^	IHM during vaccination period; % (n/N)^a^	OR (95% CI)	*p*-value*
Total	16.2 (53,304/329,690)	11.5 (22,291/194,624)	0.67 (0.66–0.68)	<0.001
Sex				
Male	17.2 (31,293/181,765)	12.2 (13,490/110,611)	0.67 (0.65–0.68)	<0.001
Female	14.9 (22,011/147,922)	10.5 (8,801/84,012)	0.67 (0.65–0.69)	<0.001
Age, range (years)				
18–39	1.0 (236/24,071)	0.7 (167/24,327)	0.70 (0.57–0.85)	<0.001
40–59	3.2 (2,682/82,827)	3.1 (1,727/56,369)	0.94 (0.89–1.00)	0.071
60–79	14.5 (18,341/126,673)	12.4 (8,989/72,784)	0.83 (0.81–0.85)	<0.001
≥80	33.3 (32,045/96,119)	27.7 (11,408/41,144)	0.76 (0.75–0.79)	<0.001
Hypertension				
No	11.1 (18,844/169,668)	7.4 (8,170/10,9,875)	0.64 (0.63–0.66)	<0.001
Yes	21.5 (34,460/160,022)	16.7 (14,121/84,749)	0.73 (0.71–0.74)	<0.001
Dyslipidemia				
No	15.1 (34,497/22,7,883)	10.5 (14,596/139,362)	0.65 (0.64–0.67)	<0.001
Yes	18.5 (18,807/101,807)	13.9 (7,695/55,262)	0.71 (0.69–0.73)	<0.001
Diabetes mellitus				
No	14.5 (36,930/254,153)	10.1 (15,435/152,446)	0.66 (0.65–0,76)	<0.001
Yes	27.7 (16,374/75,537)	16.3 (6,856/42,178)	0.70 (0.68–0.72)	<0.001
Obesity				
No	16.4 (47,948/292,613)	11.7 (19,275/164,587)	0.68 (0.66–0.68)	<0.001
Yes	14.4 (5,356/37,077)	10.0 (3,016/30,037)	0.66 (0.63–0.69)	<0.001
CKF				
No	14.2 (41,532/292,475)	9.8 (17,270/175,750)	0.66 (0.65–0.67)	<0.001
Yes	31.6 (11,772/37,215)	26.6 (5,021/18,874)	0.78 (0.75–0.81)	<0.001
NDDs				
No	14.5 (43,770/301,538)	10.5 (19,384/184,006)	0.69 (0.68–0.71)	<0.001
Yes	33.9 (9,534/28,152)	27.4 (2,907/101,618)	0.74 (0.70–0.77)	<0.001
Heart failure				
No	14.5 (43,792/302,699)	9.9 (17,969/181,062)	0.65 (0.64–0.66)	<0.001
Yes	35.2 (9,512/26,991)	31.9 (4,322/13,562)	0.86 (0.23–0.89)	<0.001
IHD				
No	15.2 (46,360/304,605)	10.6 (19,332/181,950)	0.66 (0.65–0.71)	<0.001
Yes	27.7 (6,944/25,085)	23.3 (2,959/12,674)	0.79 (0.76–0,84)	<0.001
COPD				
No	15.6 (48,088/309,112)	10.9 (20,081/183,913)	0.66 (0.65–0,68)	<0.001
Yes	25.3 (5,216/20,578)	20.6 (2,210/10,711)	0.77 (0.73–0.81)	<0.001
Neoplasm				
No	15.5 (48,868/314,560)	10.8 (20,287/187,010)	0.66 (0.65–0.67)	<0.001
Yes	29.3 (4,436/15,130)	26.3 (2,004/7,614)	0.86 (0.81–0.92)	<0.001
CVD				
No	15.7 (50,044/319,263)	11.1 (20,997/189,700)	0.67 (0.66–0.68)	<0.001
Yes	31.3 (3,260/10,427)	26.3 (1,294/4,924)	0.78 (0.72–0.84)	<0.001
Lymphoma				
No	16.0 (51,987/325,074)	11.2 (21,468/191,672)	0.66 (0.65–0.67)	<0.001
Yes	28.5 (1,317/4,616)	27.9 (823/2,952)	0.96 (0.87–1.07)	0.548
Transplant				
No	16.1 (52,853/327,291)	11.4 (21,946/192,914)	0.66 (0.65/0.69)	<0.001
Yes	18.8 (451/2,399)	20.2 (345/1,710)	1.09 (0.93–1.28)	0.280
Hemodialysis				
No	16.1 (52,647/327,212)	11.4 (22,054/193,574)	0.67 (0.66–0.68)	<0.001
Yes	26.5 (657/2,478)	22.6 (237/1,050)	0.80 (0.68–0.95)	0.014
CLD				
No	16.1 (52,946/328,292)	11.4 (22,114/193,826)	0.67 (0.66–0.68)	<0.001
Yes	25.6 (358/1,398)	22.2 (177/798)	0.82 (0.67–1.01)	0.078
Leukemia				
No	16.1 (53,023/328,859)	11.4 (22,205/194,272)	0.67 (0.66–0.68)	<0.001
Yes	33.8 (281/831)	24.4 (86/352)	0.63 (0.48–0.84)	0.002
HIV				
No	16.2 (52,316/328,890)	11.5 (22,247/194,127)	0.67 (0.66–0.68)	<0.001
Yes	11.8 (88/800)	8.9 (44/94)	0.78 (0.53–1.15)	0.221
ICU Admission				
No	14.7 (44,386/301,536)	9.4 (151,429/167,181)	0.60 (0.59–0.64)	<0.001
Yes	31.7 (8,908/28,099)	24.0 (6,506/27,125)	0.68 (0.65–0.71)	<0.001

a The results are presented as percentages (number of events per sample size).

*The statistical test used was the chi-square test. %, percentage; n. number of cases, N, total number; IHM, in-hospital mortality; CKF, chronic kidney failure; IHD, ischemic heart disease; CVD, cerebrovascular disease; CLD, chronic liver disease; HIV: human immunodeficiency virus; NDDs, neurodegenerative disorders; COPD, chronic obstructive pulmonary disease.

The IHM increased progressively with each age stratum, from 0.8% in patients aged 18–39 years to 31.7% in patients aged ≥80 years. There was a decrease in IHM across all age groups during the vaccination period, with the most pronounced reduction observed in patients over 80 years old (OR: 0.76, 95% CI: 0.75–0.79, *p*-value <0.001; Table 3). IHM was greater in males than in females (15.3% vs. 13.3%, *p*-value <0.001), with a similar reduction observed in both sexes between the prevaccination and vaccination study periods (for males, from 17.2 to 12.2%, and for females, from 14.9 to 10.5%). The IHM decreased during the vaccination period for all the comorbidities analyzed, as well as for patients admitted to the ICU (OR: 0.60, 95% CI: 0.59–0.61, *p*-value <0.001).

### 3.4 Mortality and risk factors in the overall period

**Figure 1:**
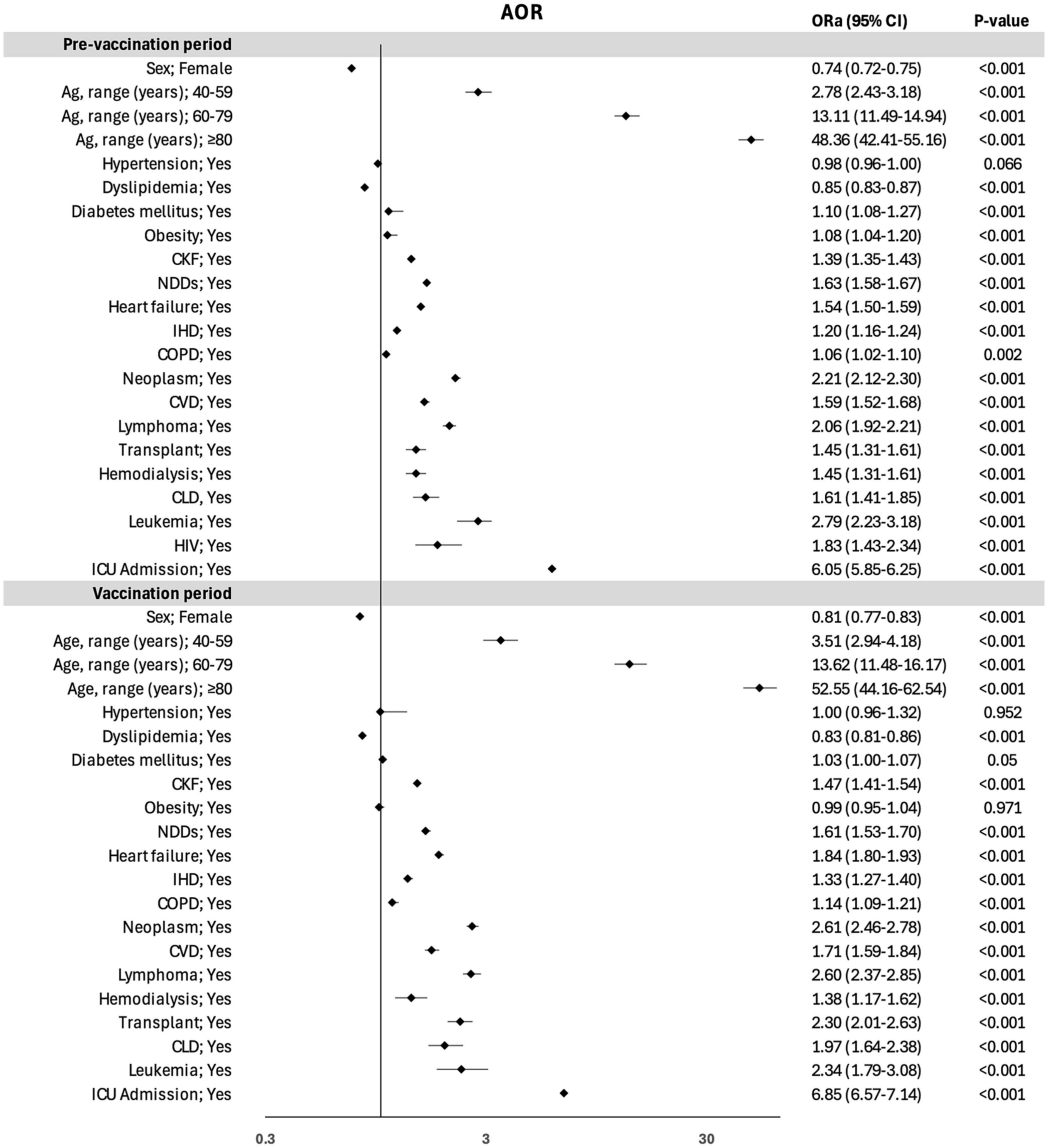
Risk factors for in-hospital mortality in the overall Variables were included as covariates if they were significantly associated according to simple models. The 95% confidence intervals (CIs) of the odds ratios were adjusted (AOR) for multiple testing.

The univariate analysis of risk factors associated with IHM over the study period is presented in Table 5. The results of the multivariate analysis—variables were included as covariates if they were significantly associated according to simple models—with the AOR values of IHM are illustrated in Figure 1. AOR of IHM during the vaccination period was 0.71 (95% CI: 0.70–0.73). The primary factor associated with IHM was age, with an AOR of 51.67 (95% CI: 46.7–57.17) for patients aged ≥80 years compared with those aged 18–39 years. Admission to the ICU was also associated with a greater AOR (6.30, 95% CI: 6.14–6.47). Female patients had a lower risk of IHM (AOR: 0.75, 95% CI: 0.74–0.77). Most of the comorbidities analyzed were associated with increased mortality; notably, patients with leukemia, neoplasms, and lymphoma had higher AOR values of 2.60 (95% CI: 2.26–2.9), 2.33 (95% CI: 2.25–2.41), and 2.24 (95% CI: 2.12–2.37), respectively. Hypertension was not associated with IHM, and dyslipidemia was associated with a lower risk.

**Table 5:** Risk factors for in-hospital mortality overall.

	IHM; % (n/N)^a^	OR (95% CI)	*p* value*
Period			
Prevaccination	16.2 (53,304/329,690)	1	
Vaccination	11.5 (22,291/194,624)	0.67 (0.66–0.68)	<0.001
Sex			
Male	15.3 (44,783/292,376)	1	
Female	13.3 (30,812/231,934)	0.85 (0.34–0.86)	<0.001
Age, range (years)			
18–39	0.8 (403/48,398)	1	
40–59	3.2 (4,409/139,196)	3.89 (3.52–4.31)	<0.001
60–79	13.7 (27,330/199,457)	18.91 (17.12–20.87)	<0.001
≥80	31.7 (43,453/137,263)	55.16 (49.98–60.8)	<0.001
Hypertension			
No	9.7 (27,014/279,543)	1	
Yes	19.8 (48,581/244,771)	2.23 (2.28–2.35)	<0.001
Dyslipidemia			
No	13.4 (49,093/367,245)	1	
Yes	16.9 (16,502/157,069)	1.31 (1.29–1.33)	<0.001
Diabetes mellitus			
No	12.9 (52,365/406,599)		
Yes	19.7 (23,230/117,715)	1.66 (1.63–1.69)	<0.001
Obesity			
No	14.7(67,223/457,200)	1	
Yes	12.5 (8,372/67,114)	0.83 (0.81–0.85)	<0.001
CKF			
No	12.6 (58,802/468,225)	1	
Yes	29.9 (16,793/56,089)	2.97 (2.91–3.02)	<0.001
NDDs			
No	13.0 (63,154/185,544)	1	
Yes	32.1 (12,441/38,770)	3.16 (3.09–3.22)	<0.001
Heart failure			
No	12.8 (61,761/483,761)	1	
Yes	34.1 (13,834/40,553)	3.54 (3.46–3.67)	<0.001
IHD			
No	13.5 (65,697/486,555)	1	
Yes	26.2 (9,903/37,759)	2.27 (2.22–2.33)	<0.001
COPD			
No	13.8 (68,169/493,025)	1	
Yes	23.7 (7,426/31,289)	1.93 (1.88–1.99)	<0.001
Neoplasm			
No	13.8 (69,155/501,570)	1	
Yes	28.3 (6,440/27,744)	2.47 (2.39–2.54)	<0.001
CVD			
No	14.0 (71,041/508,963)	1	
Yes	29.7 (4,554/15,351)	2.60 (2.50–2.69)	<0.001
Lymphoma			
No	14.2 (73,455/516,746)	1	
Yes	28.3 (2,140/7,568)	2.38 (2.26–2.53)	<0.001
Transplant			
No	14.4 (74,799/520,205)	1	
Yes	19.4 (796/4,109)	1.43 (1.32–1.54)	<0.001
Hemodialysis			
No	14.3 (74,701/520,786)	1	
Yes	25.3 (894/3,528)	2.03 (1.87–2.19)	<0.001
CLD			
No	14.4 (75,060/522,118)	1	
Yes	24.4 (535/2,196)	1.91 (1.74–2.11)	<0.001
Leukemia			
No	14.4 (75,228/523,131)	1	
Yes	31.0 (367/1,183)	2.67 (2.36–3.03)	<0.001
HIV			
No	14.4 (75,463/523,017)	1	
Yes	10.2 (132/1,297)	0.67 (0.56–0.80)	<0.001
ICU Admission			
No	12.8 (60,138/468,717)	1	
Yes	27.9 (15,414/55,224)	2.63 (2.57–2.68)	<0.001

a The results are presented as percentages (number of events per sample size).

*The statistical test used was the chi-square test. %, percentage; n, number of cases; N, total number; IHM, in-hospital mortality; CKF, chronic kidney failure; IHD, ischemic heart disease; CVD, cerebrovascular disease; CLD, chronic liver disease; HIV, human immunodeficiency virus; NDDs, neurodegenerative disorders; COPD, chronic obstructive pulmonary disease.

### 3.5 Mortality and risk factors across the prevaccination and vaccination study periods

**Figure 2:**
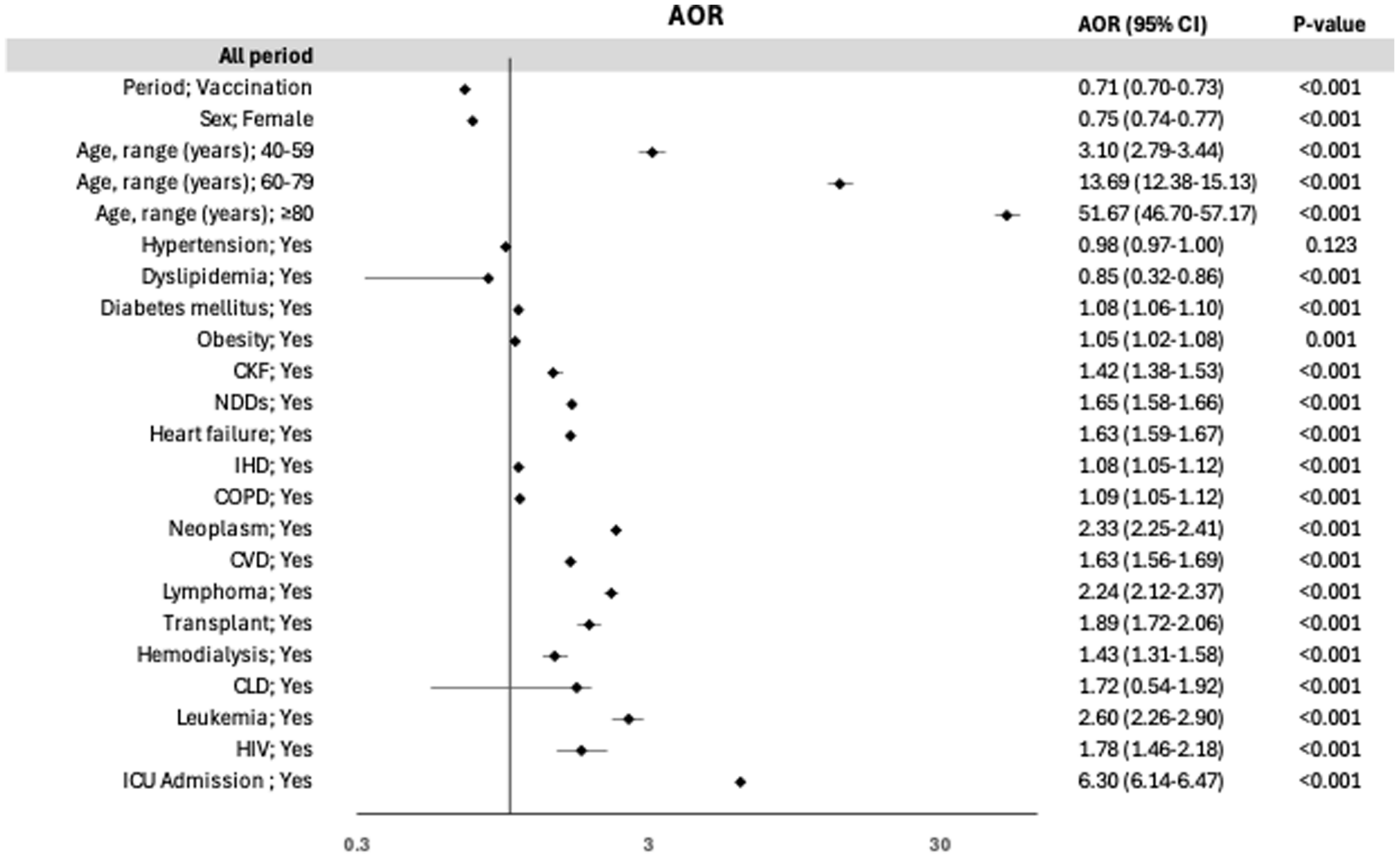
Risk factors for in-hospital mortality in the prevaccination and vaccination study periods. Variables were included as covariates if they were significantly associated according to simple models. The 95% confidence intervals (CIs) of the odds ratios were adjusted (AOR) for multiple testing.

Table 6 provides an analysis of the IHM risk factors stratified for each specific period (prevaccination / vaccination). The results of the multivariate analysis—variables were included as covariates if they were significantly associated according to simple models—with the AOR values of IHM stratified for each specific period are illustrated in Figure 2. Age was the principal factor associated with IHM. Older patients (aged >80 years) had similar AORs in both periods (AOR of 48.3 [95% CI: 42.4–55.1] for the first period and 52.2 [95% CI: 44.1–62.5] for the second period). Female sex was associated with a 26% reduction in mortality in prevaccination period and a 19% reduction in mortality in vaccination period.

**Table 6:** Risk factors for in-hospital mortality in the prevaccination and vaccination periods.

	Prevaccination period			Vaccination period		
	IHM; % (n/N)	OR (95% CI)	*p* value	IHM; % (n/N)	OR (95% CI)	*p* value
Period	16.2 (53,304/329,690)			11.5 (22,291/194,624)		
Sex						
Male	17.2 (31,293/181,765)	1		12.2 (13,490/10,611)	1	
Female	14.9 (22,011/147,922)	0.84 (0.82–0.85)	<0.001	10.5 (8,801/84,012)	0.84 (0.82–0.87)	<0.001
Age, range (years)						
18–39	1.0 (236/24,071)	1		0.7 (167/24,327)	1	
40–59	3.2 (2,682/82,827)	3.38 (0.29–3.86)	<0.001	3.1 (1,727/56,369)	4.57 (1.89–5.63)	<0.001
60–79	14.5 (18,341/126,673)	17.10 (15.02–19.46)	<0.001	12.4 (8,989/72,784)	20.38 (17.47–23.77)	<0.001
≥80	33.3 (32,045/96,119)	50.51 (44.40–57.46)	<0.001	27.7 (11,408/41,144)	55.50 (47.59–64.72)	<0.001
Hypertension						
No	11.1 (18,844/169,668)	1		7.4 (8,170/109,875)	1	
Yes	21.5 (34,460/160,022)	2.19 (2.16–2.24)	<0.001	16.7 (14,121/84,749)	2.49 (2.41–2.59)	<0.001
Dyslipidemia						
No	15.1 (34,497/227,883)	1		10.5 (14,596/139,362)	1	
Yes	18.5 (18,807/101,807)	1.27 (1.24–1.29)	<0.001	13.9 (7,695/55,262)	1.38 (1.34–1.42)	<0.001
Diabetes mellitus						
No	14.5 (36,930/254,153)	1		10.1 (15,435/152,446)	1	
Yes	21.7 (16,374/75,537)	1.63 (1.59–1.66)	<0.001	16.3 (6,856/42,178)	1.72 (1.67–1.78)	<0.001
Obesity						
No	16.4 (47,948/292,613)	1		9.8 (17,270/175,750)	1	
Yes	14.4 (5,356/37,007)	0.86 (0.84–0.89)	<0.001	26.6 (5,021/18,874)	3.33 (3.21–3.44)	<0.001
CKF						
No	14.2 (41,532/292,475)	1		11.7 (19,275/164,587)		
Yes	31.6 (11,772/37,215)	2.79 (2.72–2.86)	<0.001	10.0 (3,016/30,037)	0.84 (0.80–0.88)	<0.001
NDDs						
No	14.5 (43,770/301,538)	1		10.5 (19,384/184,006)	1	
Yes	33.9 (9,534/28,152)	3.02 (2.94–3.09)	<0.001	27.4 (2,907/10,618)	3.02 (3.06–3.35)	<0.001
Heart failure						
No	14.5 (43,792/302,699)	1		9.9 (17,969/181,062)	1	
Yes	35.2 (9,512/26,991)	3.21 (3.13–3.30)	<0.001	31.9 (4,322/13,562)	4.24 (4.08–4.41)	<0.001
IHD						
No	15.2 (46,360/304,605)	1		10.6 (19,332/181,950)	1	
Yes	27.7 (6,944/25,085)	2.13 (2.07–2.19)	<0.001	23.3 (2,959/12,674)	2.56 (2.45–2.67)	<0.001
COPD						
No	15.6 (48,088/309,112)	1		10.9 (20,081/183,913)	1	
Yes	25.3 (5,216/20,578)	1.84 (1.78–1.90)	<0.001	20.6 (2,210/10,711)	2,12 (2.02–2.22)	<0.001
Neoplasm						
No	15.5 (48,868/314,560)	1		10.8 (20,287/187,010)	1	
Yes	29.3 (4,436/15,130)	2.25 (2.17–2.34)	<0.001	26.3 (2,004/7,614)	2.93 (2.78–3.09)	<0.001
CVD						
No	15.7 (50,044/319,263)	1		11.1 (20,997/189,700)	1	
Yes	31.3 (3,260/104,427)	2.45 (2.34–2.55)	<0.001	26.3 (1,294/4,924)	2.86 (2.68–3.06)	<0.001
Lymphoma						
No	16.0 (51,987/325,074)	1		11.2 (21,468/191,672)	1	
Yes	28.5 (1,317/4,616)	2.10 (1.96–2.23)	<0.001	27.9 (823/2,952)	3.06 (2.82–3.36)	<0.001
Transplant						
No	16.1 (52,853/327,291)	1		11.4 (22,054/193,574)	1	
Yes	18.8 (451/2,399)	1.20 (1.08–1.33)	<0.001	22.6 (237/1,050)	2.26 (1.96–2.62)	<0.001
Hemodialysis						
No	16.1 (52,647/327,212)	1		11.4 (21,946/192,914)	1	
Yes	26.5 (657/2,478)	1.88 (1.72–2.06)	<0.001	20.2 (345/1,710)	1.97 (1.74–2.21)	<0.001
CLD						
No	16.1 (52,946/328,292)	1		11.4 (22,114/193,826)	1	
Yes	25.6 (358/1,398)	1.79 (1.58–2.02)	<0.001	22.2 (177/798)	2.21 (1.87–2.61)	<0.001
Leukemia						
No	16.1 (53,023/328,859)	1		11.4 (22,205/194,272)	1	
Yes	33.8 (281/831)	2.66 (2.30–3.07)	<0.001	24.4 (86/352)	2.50 (1.96–3.19)	<0.001
HIV						
No	16.2 (53,216/328,890)	1		11.5 (2,224/194,127)	1	
Yes	11.0 (88/800)	0.64 (0.51–0.79)	<0.001	8.9 (44/497)	0.75 (0.55–1.02)	0.068
ICU Admission						
No	14.7 (44,386/30,1,536)	1		10.6 (19,332/181,950)	1	
Yes	31.7 (8,908/28,099)	2.69 (2.61–2.76)	<0.001	23.3 (2,959/12,674)	2.56 (2.45–2.67)	<0.001

%, percentage; n, number of cases; N, total number; IHM, in-hospital mortality; CKF, chronic kidney failure; IHD, ischemic heart disease; CVD, cerebrovascular disease; CLD, chronic liver disease; HIV: human immunodeficiency virus; NDDs, neurodegenerative disorders; COPD: chronic obstructive pulmonary disease.

Admission to the ICU showed similar increased AOR values in both periods. Most of the comorbidities analyzed presented consistent AOR values across both periods. Patients with leukemia, neoplasms, and lymphoma presented the highest AOR values, which were very similar between the two periods. However, the AOR for transplant patients was greater in the second period, with AORs of 2.3 (95% CI: 2.01–2.63) vs. 1.45 (95% CI: 1.31–1.61; Figure 2).

Hypertension was not associated with IHM in either period, and obesity and HIV infection were identified as risk factors only in the first period. Dyslipidemia was a protective factor against mortality in both periods, with similar AOR values for the first period (0.85 for the first period [95% CI: 0.83–0.87] and 0.83 [95% CI: 0.81–0.86] for the second period; Figure 2).

## 4 Discussion

The findings from this large registry-based study demonstrate that COVID-19 outcomes among hospitalized patients have considerably improved over the first 2 years of the pandemic, The risk of IHM was 29% lower for patients during the vaccination period compared to the prevaccination period, after adjusting for sex, age groups, comorbidities, and ICU admission The risk factors for IHM were consistent across both periods, with age and cancer identified as primary risk factors, whereas.

hypertension was notably not a risk factor. Obesity and HIV infection were only risk factors in the prevaccination period after adjustment for other variables. Female sex and dyslipidemia were protective factors. In our study, the interaction between the risk-factors and prevaccination and vaccination study periods in the IHM were controlled by inclusion these risk factors were included in the multivariate analysis.

The reduction in mortality between the two study periods (a 0.29% decrease in deaths) aligns with findings from published studies in the literature. The IHM in 2021 was comparable to rates reported in other European countries, such as England (26), Italy (27), France (13), and the Netherlands (28), as well as in the United States (26). Several factors contributed to this improvement, including virus variants, the introduction of more effective COVID-19 treatments, and intensive vaccination programs (12, 13, 26–30).

Vaccination in Spain was widely implemented in the early months of 2021, covering more than 75% of the population by August 2021 (25), and has been reported as the main factor associated with decreased mortality in most published cohorts per example in England (12), France (13, 31), Spain (11, 32) Israel (33), as in our study.

In Spain, the first nationwide study reported by García-Carretero et al. (20) analyzed the number of hospitalizations, ICU admissions, and deaths due to COVID-19 over 2 years (2020–2021), but risk factors related to disease severity were not analyzed in this cohort. These findings are similar to those presented in our study, which revealed a reduction in hospitalization and mortality rates and an increase in ICU admissions in the second year. The observed increase in ICU admissions in different cohorts has been linked to the characteristics of the hospitalized population during the first year, particularly their older age and higher comorbidity burden, which may have contributed to the limitations in therapeutic efforts or resource allocation associated with the first wave rather than indicating a greater severity of infection (32).

We analyzed the most prevalent comorbidities recorded in the SNSSHD between the between the prevaccination and vaccination study periods. In 2021, hospitalized patients were younger and had fewer comorbidities than they did in the previous year, likely due to the initiation of vaccination in older populations and those with significant comorbidities (31).

The risk factors for IHM were similar between the prevaccination and vaccination study periods and were consistent with those reported since the onset of the pandemic (33); however, the degree of impact differed among the various associated factors.

Age was the main risk factor in our study, with patients over 80 years of age exhibiting high mortality rates regardless of the all study period, prevaccination and vaccination study periods. Although age is regarded as a key prognostic factor in people hospitalized with COVID-19, a recent study that included 10,551 hospitalizations in Spain suggested that, when measured exhaustively, the comorbidity burden better explains the greater risk of critical illness (ICU admission, need for invasive mechanical ventilation, or in-hospital death) than chronological age does (34). Our analysis, which considers only mortality, supports the association with mortality in very older patients. Including ICU admission as a variable may introduce a confounding factor in the analysis of clinical outcomes.

In terms of comorbidities, arterial hypertension, initially identified as a risk factor (33), was not associated with increased severity in this Spanish cohort. In contrast, other comorbidities were associated with IHM, with the strongest associations observed for factors related to immunosuppression, such as solid or hematological neoplasms. This is consistent with population-based series, where immunosuppression continues to be a risk factor throughout different waves of the pandemic (35, 36). A systematic review assessed the existing data describing the efficacy of COVID-19 vaccination in protecting immunocompromised individuals against breakthrough infections and severe COVID-19 outcomes (37). These individuals are also more likely to experience severe complications from these breakthrough infections and do not mount the same immune response following vaccination.

According to a recent meta-analysis, obesity was associated with a 34% relative increase in the odds of mortality (*p*-value = 0.002), although there was considerable heterogeneity in the results (38). Only one report specifically compared vaccinated and unvaccinated obese patients, revealing a trend toward lower mortality in the vaccinated group, which is consistent with the findings of our study.

In addition to vaccination as the main protective factor, only dyslipidemia and female sex were identified as factors associated with lower mortality in the prevaccination and vaccination study periods. Patients with a recorded diagnosis of dyslipidemia are likely receiving statins, which could explain the protective role observed. This finding is supported by a recent meta-analysis of seven randomized clinical trials that revealed that statins reduce case fatality rates in patients hospitalized with COVID-19 (39).

As extensively documented in the literature and corroborated by our findings, females experienced lower mortality rates related to COVID-19 than males did (9, 10, 40). Sex-related differences, such as immune responses and hormone levels, could be related to the mortality rates of patients with COVID-19 (41).

The major strength of our study is the use of a comprehensive medical-administrative database that includes over half a million COVID-19 hospitalizations across Spain over 2 years, allowing for robust analysis of in-hospital mortality trends and associated risk factors. To our knowledge, this is one of the largest studies to analyze COVID-19 hospitalizations and IHM between the prevaccination and vaccination study periods, adjusting for age, sex, and comorbidities. This large population study allows us to identify risk factors associated with COVID-19 mortality, which are consistent throughout the evolution of the pandemic and are related to vaccination, SARS-CoV-2 variants, or therapeutic strategies.

The limitations of our study are inherent to the use of medico-administrative databases and include potential biases (16, 17) associated with reliance on ICD-10 coding, as patient medical histories were not accessible, potentially impacting data accuracy. Additionally, the lack of detailed information on treatments (such as remdesivir, steroids, or ventilation), patient clinical status, lifestyle behaviors (smoking habits, weight, alcohol consumption, glycaemic values, etc.), laboratory and radiological findings, and vaccination status limits our ability to evaluate specific therapeutic interventions or outcomes. Other limitation is each hospital admission is considered a new patient, and readmissions are treated as separate cases. Therefore, findings derived from healthcare administrative databases should be interpreted with caution due to these potential biases (16, 17).

## 5 Conclusion

In conclusion, the overall impact of COVID-19 hospitalizations over the first 2 years of the pandemic resulted in more than half a million admissions. The risk of in IHM was 29% lower for patients during the vaccination period compared to the prevaccination period, after adjusting for sex, age groups, comorbidities, and ICU admission, with similar reductions observed across both sexes, age groups, and patients with comorbidities. The risk factors for IHM were consistent between the prevaccination and vaccination study periods, with age and solid/hematologic cancers being the main risk factors. The vaccination period, female sex, and dyslipidemia served as protective factors against in-hospital mortality. The COVID-19 hospitalizations recorded in SNSSHD provide critical insights into the burden of severe COVID-19 in Spain. Analysis of these data is invaluable for documenting the severity of COVID-19 hospitalizations and identifying risk factors associated with mortality across different scenarios.

## Authors note

Jose-Manuel Ramos-Rincon affirms that this manuscript is an honest, accurate, and transparent account of the study being reported, that no important aspects of the study have been omitted, and that any discrepancies from the study as planned (and, if relevant, registered) have been explained.

## Data Availability

The raw data supporting the conclusions of this article will be made available by the authors, without undue reservation.
