# Supplementary material for: A national population-based study of mortality and risk factors in COVID-19-hospitalized patients in Spain (2020–2021)
Source: Front Public Health. 2025 Feb 4;13:1488283. doi: 10.3389/fpubh.2025.1488283 (PMC11841506; doi:10.3389/fpubh.2025.1488283)
Supplement: Supplementary file 1 [file Table_1.docx]

**Table 1S. List of International Classification of Diseases, Tenth Revision, Clinical Modification diagnosis (ICD-10-CM) codes used in the study.**

| **Disease** | **ICD 10 CM** |
| --- | --- |
| COVID- 19 | B34.2; B97.29; J12.82; J12.8; Z20.828  U07.1; |
| **Comorbidities** |  |
| Hypertension | I10; I11; I12; I13; I15 |
| Dyslipidemia | E78 |
| Diabetes mellitus | E10; E11 |
| Obesity | E66 |
| Chronic kidney disease | N18 |
| Neurodegenerative disorders | F01; F02; F03; G30; G31; G45; G46 |
| Heart failure | I50 |
| Ischemic heart disease | I21; I22; I23; I24; I25 |
| Chronic obstructive pulmonary disease. | J44 |
| Neoplasm | C01; C02; C03; C04; C05; C06; C07; C08; C09; C10; C11; C12; C13; C14; C15; C16; C17; C18; C19; C20; C21; C22; C23; C24; C25; C26; C27; C28; C29; C30; C31; C32; C33; C34; C35; C36; C37; C38; C39; C40; C41; C42; C43; C44; C45; C46; C47; C48; C49; C50; C51; C52; C53; C54; C55; C56; C57; C58; C59; C60; C61; C62; C63; C64; C65; C66; C67; C68; C69; C70; C71; C72; C73; C74; C75; C76; C77; C78; C79; C80 |
| Cerebrovascular diseases | I60; I61; I62; I63; I64; I65; I66; I68; I69 |
| Lymphoma | C81; C82; C83; C84; C85; C86 ; C87 ; C88 ; C89 ; C90; C91 |
| Transplant | Z94 |
| Hemodialysis | Z99.2 |
| Chronic Liver Disease | K74 |
| Leukemia | C92; C93; C94; C95; C96 |
| Human Immunodeficiency virus infection | B20 |
